# Eosinophilic gastritis in adult complicated by gastric ulcer perforation

**DOI:** 10.1093/jscr/rjae113

**Published:** 2024-03-06

**Authors:** Joseph A Tharakan, Julianna P O’Toole, Harmeet K Kharoud, Mark L Bunker, Jennifer Y Chen, Shinichiro Yokota

**Affiliations:** Department of Surgery, Allegheny Health Network, Pittsburgh, PA 15212, United States; Department of Surgery, Allegheny Health Network, Pittsburgh, PA 15212, United States; Department of Pathology, Allegheny Health Network, Pittsburgh, PA 15212, United States; Department of Pathology, Allegheny Health Network, Pittsburgh, PA 15212, United States; Department of Surgery, Allegheny Health Network, Pittsburgh, PA 15212, United States; Department of Surgery, Allegheny Health Network, Pittsburgh, PA 15212, United States; Department of Surgery, Division of Abdominal Transplantation, Stanford University School of Medicine, 750 Welch Road, Suite 319, Palo Alto, CA 94304, United States

**Keywords:** eosinophilic gastritis, gastric ulcer perforation, laparotomy

## Abstract

Eosinophilic gastritis is a rare type of eosinophilic gastrointestinal diseases. Patients with eosinophilic gastritis usually present with symptoms such as nausea, emesis, abdominal pain, and weight loss. In severe cases, patients can suffer rare complications such as gastric outlet obstruction and spontaneous perforation. Here, we present the case of a young adult male who presented with acute onset abdominal pain for 1 day. The patient was found to have significant mural thickening of gastric antrum with pneumoperitoneum on abdominal CT scan, consistent with a perforated gastric ulcer. The patient underwent exploratory laparotomy and required modified graham patch repair. The diagnosis of eosinophilic gastritis was made based on the pathology review of intraoperative endoscopic biopsy specimens.

## Introduction

Eosinophilic gastrointestinal diseases (EGIDs) are chronic, immune-mediated processes characterized by eosinophilic infiltration of a certain segment (s) in the gastrointestinal (GI) tract without secondary causes of eosinophilia [1]. Eosinophilic esophagitis (EoE) is the most common and the most studied type of EGIDs [2]. In contrast, eosinophilic gastritis is an unusual type of EGIDs that only affects the stomach and remains understudied because of its rarity. Patients with eosinophilic gastritis usually present with nausea, abdominal pain, and weight loss due to malnutrition. Gastric ulcer perforation is a rare complication associated with eosinophilic gastritis and there are only a few reports available in the literature [3]. We present a case of a young adult male patient with eosinophilic gastritis who developed gastric ulcer perforation requiring exploratory laparotomy.

## Case report

A young adult male with a past medical history of asthma and gastroesophageal reflux disease (GERD) presented to the Emergency Department with acute epigastric pain and nausea that started that same evening.

**Figure 1:**
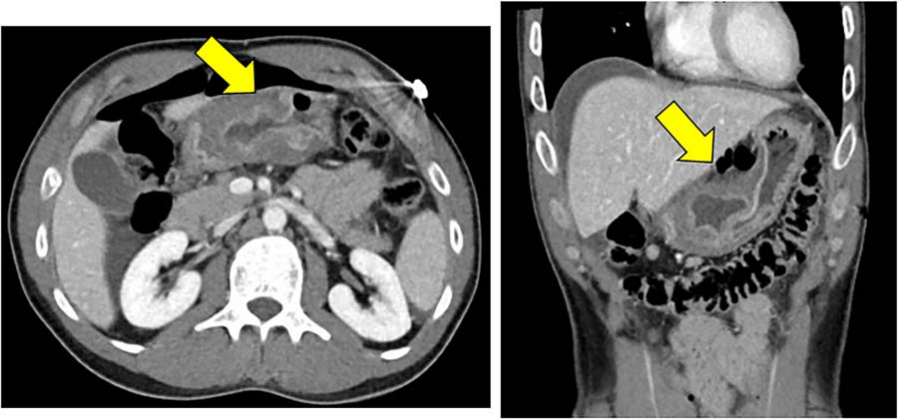
Significant mural thickening of gastric antrum with pneumoperitoneum along the anterior, superior wall, and free fluid around the liver, consistent with a perforated gastric ulcer.

On presentation, the patient was afebrile with stable vital signs other than mild tachycardia with a heart rate of 110 beats per minute. Blood work showed a leukocytosis of 12.6 K/mcL. Basic metabolic panel was unremarkable. Physical exam showed diffusely tender abdomen with guarding. A CT scan demonstrated marked thickening of gastric antrum with perforation along the anterior wall with pneumoperitoneum and free fluid, consistent with a perforated gastric ulcer (Fig. 1). The patient was taken emergently to the operating room for diagnostic laparoscopy.

In the operating room, we noticed significant purulent fluid in the entire abdominal cavity upon entry into the abdomen. We suctioned this purulent fluid and then exposed the stomach. We found a large, perforated ulcer in the antrum and noticed significant induration and nodularity surrounding the perforated ulcer. We also noted copious gastric contents were leaking through this perforation. At this point, we converted to open exploration due to significant contamination.

An upper midline incision was created to expose the distal stomach and the perforated ulcer was 1.5 cm in size. We then performed modified graham patch repair to close the ulcer primarily. We subsequently performed intraoperative esophagogastroduodenoscopy (EGD) because of a concern for malignancy and took two mucosal biopsies around the ulcer. No air leak was noted from the serosal surface of the repair at that time. We confirmed that a nasogastric tube with its tip in the stomach at the end of the case. The abdominal cavity was irrigated and a 19 Fr drain was placed before the closure of the abdomen.

The patient’s postoperative course was uncomplicated. Upon further questioning, the patient revealed that he was incidentally found to have mural thickening of his stomach several months ago when he was involved in a minor trauma accident. The patient did not sustain trauma injuries but was instructed to follow up with a gastroenterologist regarding the mural thickening of the stomach. The patient was lost to follow-up. Prior CT scan showed similar mural thickening without perforation and demonstrated the chronic nature of the disease (data not shown).

**Figure 2:**
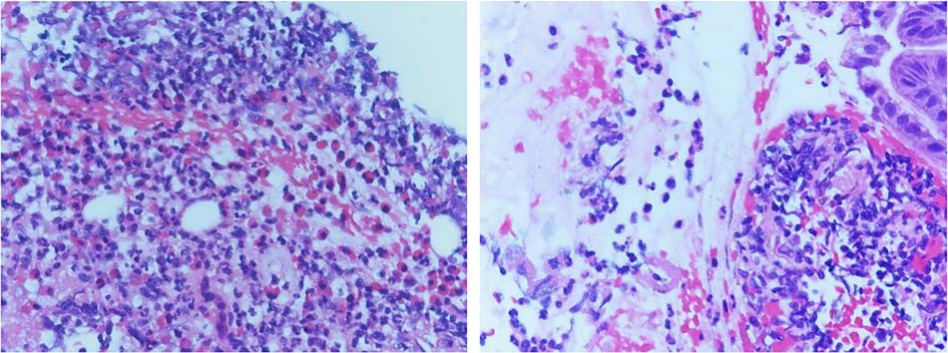
Pathology review demonstrated increased eosinophil infiltration (>20 eosinophils per 40 x High Power Field) in submucosa with fragments of gastric mucosa, consistent with eosinophilic gastritis.

The pathology report of the intraoperative biopsy showed eosinophilic gastritis (>20 eosinophils per high power field) (Fig. 2) and *Helicobacter Pylori* testing was negative. An upper GI study performed postoperative Day 5 showed no evidence of a leak from the primary repair in the stomach, and the patient was started on a clear liquid diet. The patient was subsequently discharged on postoperative Day 7. The patient was seen in the surgery clinic on postoperative Day 13 and was recovering appropriately. The patient followed up with the gastroenterology clinic and was placed on a 6-food elimination diet and proton pump inhibitor with the plan for a follow-up outpatient EGD.

## Discussion

EGIDs are a group of GI diseases that present with GI symptoms associated with eosinophilic infiltration of a certain segment(s) in the GI tract in the absence of other causes of eosinophilia [4]. Although the exact mechanism of this disease is unknown, it is suspected that these disorders are at least partly because of allergic reactions to certain foods [5].

The most commonly affected segment in the GI tract is the esophagus, which is termed EoE, with its estimated prevalence of 57 cases/100000 in the USA [6]. Other EGIDs are much less common in comparison; the prevalence of eosinophilic gastritis, eosinophilic gastroenteritis, and eosinophilic colitis was 6.7, 8.2, and 3.5 cases/100000 [5]. Eosinophilic gastroenteritis has the highest prevalence among children age < 5 years [5]. Because of historically low incidence rates of non-EOE EGIDs, these disease processes remained understudied [7]. However, non-EoE EGIDs are being diagnosed at an increasing rate and the American Gastroenterological Association recently published international consensus recommendations for nomenclature for EGIDs for both clinical and research use in 2022, given the increasing clinical importance of non-EOE EGIDs [1, 4, 5].

GI symptoms associated with non-EoE EGIDs are nausea, vomiting, diarrhea, abdominal pain, and weight loss [1, 7]. In severe cases, patients can suffer complications including gastric outlet obstruction, small bowel obstruction, intussusception, and spontaneous perforation [8–10]. Spontaneous perforation is a very rare complication of non-EoE EGIDs. Our search found <15 case reports with perforation distal to the esophagus in the literature (between 1977 and 2023) [3, 8, 9, 11–14]. Only three patients had gastric perforation and one patient was an adult; the present case is the second adult patient who had gastric ulcer perforation of eosinophilic gastritis.

In the present case, the patient is unique for two reasons: (i) the patient had a CT scan several months before the episode of gastric perforation, demonstrating the chronic nature of eosinophilic gastritis, and (ii) the patient underwent intraoperative EGD and biopsy because of significantly edematous and nodular appearance of the gastric wall, which allowed us to reach the diagnosis of eosinophilic gastritis without further delay. Given the increasing clinical importance, clinicians must include eosinophilic gastritis in differential diagnoses upon examining a patient who presents with acute or chronic abdominal pain.

## Data Availability

The data underlying this article will be shared on reasonable request to the corresponding author.
